# Capability of TiO_2_ and Fe_3_O_4_ nanoparticles loaded onto Algae (*Scendesmus sp.*) as a novel bio-magnetic photocatalyst to degration of Red195 dye in the sonophotocatalytic treatment process under ultrasonic/UVA irradiation

**DOI:** 10.1038/s41598-023-45274-1

**Published:** 2023-10-24

**Authors:** Wahid Zamani, Saeedeh Rastgar, Aliakbar Hedayati

**Affiliations:** 1 Department of Environmental Science, Faculty of Natural Resources, University of Kurdistan, Sanandaj, 15175-66177 Iran; 2 Department of Environmental Sciences, Faculty of Fisheries and Environmental Sciences, Gorgan University of Agricultural Sciences and Natural Resources, Gorgan, 49189-43464 Iran; 3 Faculty of Fisheries and Environmental Sciences, Gorgan University of Agricultural Sciences and Natural Resources Gorgan, Gorgan, 49189-43464 Iran

**Keywords:** Biochemistry, Environmental sciences, Chemistry, Engineering, Materials science

## Abstract

In this study, the magnetic photocatalyst Scendesmus/Fe_3_O_4_/TiO_2_ was synthesized, and its sonophotocatalytic properties in relation to the degradation of the Red195 dye were evaluated. Particles were characterized using a scanning electron microscope (SEM), Fourier's transform infrared spectroscopy (FTIR), X-ray powder diffraction (XRD), and a vibrating-sample magnetometer (VSM). At a pH of 5, a photocatalyst dosage of 100 mg, an initial R195 concentration of 100 mg/l, an ultrasound power of 38W, and an exposure time of 20 min, the maximum Red195 removal efficiency (100%) was achieved. After five cycles of recycling, the composite's sonophotocatalytic degradation stability for R195 remains above 95%. Experiments on scavenging indicate that electrons (h^+^) and hydroxyls (OH^-^) are indispensable decomposition agents. The removal of R195 by Scendesmus/Fe_3_O_4_/TiO_2_ is consistent with the pseudo-first-order kinetic, Freundlich, and Henderson's isotherm models, as determined by kinetic and isotherm investigations. The negative activation enthalpy of the standard (ΔH°) illuminates the exothermic adsorption mechanism. The increase in standard Gibbs activation free energy (ΔG°) with increasing temperature reveals the process is not spontaneous. As indicated by the negative value of the standard entropy of activation (ΔS°), activation of the reactants resulted in a loss of freedom.

## Introduction

The textile industry's production and disposal of effluent, which frequently contains toxic metals and biodegradation-resistant organic compounds^1^. The most widely used dyes in the textile industry are azo dyes with azo (–N=N–), hydroxyl (^•^OH), and –SO_3_H groups^2^. Due to their resilience and high solubility in aqueous solutions, it is challenging to eliminate these dyes using a microorganism-based remediation method^3^. Recently, conventional physicochemical processes such as reverse osmosis, coagulation-flocculation, adsorption, and filtration have been utilized to remove persistent pollutants from wastewater^4^. The production of secondary pollutants, high post-treatment costs, and the inability of pollutants to decompose are current issues with technologies^5^. Traditional physicochemical techniques include membrane filtration, adsorption, chemical precipitation, reverse osmosis, ion exchange, electro-dialysis, solvent extraction, chemical oxidation/reduction, and others for removing dye pollutants from contaminated water^6^.

However, current methods frequently have issues, including the production of secondary pollutants, high post-treatment costs, and the inability of pollutants to degrade. In contrast to these removal methods, advanced oxidation processes (AOPs) based on the generation of free radicals such as ^•^OH and O_2_ can mineralize a wide range of contaminants without generating sediment or hazardous residues^5,6^. In recent years, researchers have shown a great deal of interest in the AOP photocatalysis system for the treatment of industrial wastewater due to its demonstrated cost-effectiveness and non-toxic, complete mineralization^7^. By absorbing light, photocatalytic systems generate electron/hole (e^-^/h^+^) pairs that degrade contaminants in the aquatic environment by producing reactive oxygen species (such as ^•^OH and O_2_^•-^)^8^. Semiconductors composed of TiO_2_ and ZnO are the materials utilized most frequently in this process. In contrast, the disadvantages of semiconductors in real-world applications include high cost, limited affinity for aromatic hydrocarbons, simple deactivation, and rapid e^-^/h^+^ pair recombination. To address these limitations, the researchers proposed the development of photocatalysts with a high light absorption potential and suitable electron transport^7,9^.

Microalgae have recently emerged as a viable microorganism for removing contaminants from effluent. Microalgae production is not commercially viable, however, due to photosynthesis and high growth and harvesting costs^10^. It is simple to obtain and cultivate green algae (*Scenedesmus spp.*), which are abundant in all aquatic environments. Due to its low cost, ease of recovery and reuse, large contact area, and high efficiency during limited contact periods, the use of algal biomass for the biosorption of pollutants is fascinating. Carbohydrates, intercellular pores, and sulfated polysaccharides observable on the adsorbent cell partition may enhance dye biosorption from effluent^11^. The recombination of photoproduced carriers limits the photocatalytic effectiveness of *Scendesmus*/TiO_2_ in large-scale treatment^12^. Since their abundance, low toxicity, and adequate band opening, it is suggested that magnetic nanoparticles be combined with *Scendesmus* or TiO_2_ to address this issue. Combining iron with *Scendesmus*/Fe_3_O_4_/TiO_2_ is advantageous because separation by an external magnetic field is easier^6^.

Recent research has combined sonocatalysis and photocatalysis to improve the treatment of resilient wastewater as well as the photocatalyst's activity^13,14^. In the sonophotocatalytic process, microbubbles are created by absorbing energy from an Ultrasonic field, which causes high concentrations of •OH^15^. Consequently, light and US waves stimulate the photocatalyst to generate more energetic radicals. In addition to enhancing adsorption, mass transfer, and particle dispersion, the resultant cavitation also cleans the active regions of the particles by removing adhesive residues^16^.

Sono-photocatalysis based on *Scendesmus*/Fe_3_O_4_/TiO_2_ is limited due to a lack of information^17–19^. In addition, data regarding the efficacy and properties of the synthetic photocatalyst are lacking^13,19,20^. Using a variety of diagnostic techniques, *Scendesmus*/Fe_3_O_4_/TiO_2_ nanoparticles were synthesized and characterized as a novel photocatalyst. In order to determine the viability of a novel photocatalyst for the well-organized degradation of R195, the process efficiencies of sono-photodegradation were compared with those of sonolysis, photolysis, sonocatalysis, and photocatalysis systems.

The main goals of this study are to synthesize a novel structured Scendesmus/Fe_3_O_4_/TiO_2_ magnetic photocatalyst, characterize and evaluate the *Scendesmus*/Fe_3_O_4_/TiO_2_ potential in R195 removal, and optimize the effects of pH, exposure time, photocatalyst dosage, dye concentration, and US power on R195 removal using response surface methodology. Thus, the findings of this study can serve as a guide for the safe and effective remediation of various textile wastewaters.

## Materials and methods

### Prepration of algae (Scendesmus sp.)

The Caspian Sea Ecology Research Institute in Mazandaran, Iran, cultivated a sample of *Scendesmus sp* Algae. The samples were then transferred to the Gorgan University of Agriculture and Natural Resources laboratory. Using a super grinder, the microalgae were then ground as finely as possible^21^.

### Synthesis of Fe_3_O_4_ nanoparticles

For the synthesizing of Fe_3_O_4_ nanoparticles, the sol–gel method was modified. To produce a golden solution, 65 ml of ethylene glycol and 4.95 g of ferric nitrate nonahydrate were stirred for two hours at 45 °C. After 10 h of increasing the temperature of the mixture to 95 °C, gel formation was obtained^22^. The emulsion was then centrifuged and desiccated in a 105 °C furnace for twenty hours (Memmert VO 400). The material was annealed for two hours at 450 °C in a vacuum furnace (Nabertherm, R80-750/11, Germany) to produce magnetic Fe_3_O_4_ nanoparticles^6^.

### synthesis of Fe_3_O_4_/scendesmus

0.49 g of Fe_3_O_4_ nanoparticles and 3.93 g of Scendesmus were dissolved in 60 ml of ethanol. At 73 °C, one hour was spent agitating the mixture. A solution of 6 ml ethanol and 1.58 ml diethanolamine was added dropwise to the initial mixture, which was stirred with a magnetic stirrer for 10 h at 76 °C until a dark brown solution was obtained. The solid was then isolated with an external magnet and dried in a vacuum furnace (Memmert VO 400) for 24 h at 90 °C^23,24^.

### Synthesis of scendesmus/Fe_3_O_4_/TiO_2_

For one hour, a mixture of synthesized *Scendesmus*/Fe_3_O_4_ and ethanol at a concentration of 99.9% was agitated. During 10 h of heating at 150 °C, the mixture was treated with 6.16 ml of tetrabutylammonium hydroxide (TBOT) and 60 ml of ethanol. After washing the sample with methanol and distilled water, it was calcined at 450 °C for three hours^6,22^.

### Point of zero charge

The point of zero charge (PZC) of algal biosorbent was determined with minor modifications to the salt addition method. The collection of various containers containing 0.1 M NaNO_3_ concentrations. HCl and NaOH (0.1 M) solutions were used to obtain pH values ranging from 2 to 10^25^. Each vial obtained 1 g of algal biosorbent and was stirred at 160 rpm for 24 h. The pH of each filtrate was noted after its constituents. Calculating the PZC value of algal biosorbent by plotting the initial pH against pH change graph^26^.

### Sonophotocatalytic experiments

Sonophotocatalysis was utilized in a US bath containing a UVA lamp to ascertain the R195 degradation rate. A precise amount of our prepared catalyst (0.25–1 mg/l) was added to a 250 mL Erlenmeyer Pyrex containing R195 at a determined pH (between 3 and 7). Using a pH meter, NaOH, and H_2_SO_4_, the initial pH adjustment was performed^27^. To better comprehend the effect of the parameters, the reactor was exposed to US frequencies (20–50 kHz) at UV light intensities (6 W). The concentration of 1 ml samples collected at different times (10–40 min) was determined using UV–vis spectrophotometry at 550 nm. Utilizing Eqs. (1) and (2), the degradation efficiency and reaction kinetics of R195 were determined:^28^

1 $$\mathrm{R}(\mathrm{\%})=\frac{(Ci-Ce)}{(C0)}\times 100$$

2 $$ {\text{q}}_{{\text{e}}} = {\text{V}}\frac{Ci - Ce}{M} $$

q_e_ in Eq. (1) quantifies the adsorbed ions (sorbate) in milligrams per gram in the biosorbent, where the initial and final metal concentrations C_i _and C_e_ were measured by atomic absorption spectrometry; V represents the volume of the synthetic medium, and M represents the biomass of the biosorbent (g)^27,28^.

### Desorption and regeneration studies

Using the supplied methodology, the reusability of the adsorbent to desorb the dye ions and regenerate the biomass for reuse was evaluated. To conduct an effective adsorption and desorption experiment, the aforementioned substances were subjected to five cycles of cycle repetition. 0.1 M hydrochloric acid was used to desorb metal ions^29^. Desorption experiments were carried out by combining a metal-loaded adsorbent with 50 mL of desorption medium (0.1 M HCl) and agitating at 160 rpm^30^. Each cycle is dominated by sorption, followed by desorption and regeneration. The following equations characterize biosorbent desorption and regeneration performance^29,30^:

3 $$ {\text{Desorption efficiency }}\% = \left( {\text{Amount of dye ion desorbed}} \right)/{\text{Amount of dye ion adsorbed}}) \times {1}00 $$

4 $$ {\text{Photocatalyst efficiency}} = \left( {{\text{Regenerated removal capacity}}/{\text{Original removal capacity}}} \right) \times {1}00 $$

### Isotherm modeling

Isotherm models help describe the interactions between contaminants and synthetic adsorbents. Langmuir, Freundlich, Temkin, and Harkins–Jura are the adsorption isotherms most frequently used. The Langmuir isotherm model depicts monolayer adsorption on a surface with restricted sites and no intermolecular interaction^8^. For a linear fit of the Langmuir isotherm model to the experimental data, the following equation was used: *C*_*e*_*/q*_*e*_ versus *C*_*e*_ (Eq. 5).

5 $$\frac{{C}_{e}}{{q}_{e}}=\frac{1}{{q}_{m}b}+\frac{1}{{q}_{m}}{C}_{e}$$

where *q*_*e*_ is the equilibrium adsorbate concentration in the adsorbent phase (mg/g), *C*_*e*_ is the equilibrium adsorbate concentration in the aqueous phase (mg/l), and *b* is the constant associated with the free adsorption energy and the concentration at which the adsorbent reaches half saturation. *q*_*m*_ also represents the maximum absorption capacity (mg/g)^5^.

The quantity of reversible adsorption on a heterogeneous surface increases with increasing concentration (Eq. 6) according to the Freundlich adsorption isotherm^31^:

6 $$ln{q}_{e}=ln{K}_{F}+\frac{1}{n}ln{C}_{e}$$

where *q*_*m*_ is the amount of molecules adsorbed to the adsorbent surface at any given time (mg/g), *C*_*e*_ is the equilibrium concentration (mg/l), *n* and *K*_*F*_ are the Freundlich constant and Freundlich exponent (mg/g (l/g)1/n, respectively), and *C*_*e*_ is the equilibrium concentration (mg/l)^5^.

As demonstrated by the following equations (Eqs. 7 and 8), the Temkin isotherm model is dependent on interactions between the absorbent and the adsorbate and assumes the linearity of the heat of adsorption of all molecules in the layer^32^:

7 $${q}_{e}=\frac{RT}{{b}_{T}}ln{A}_{T}{C}_{e}$$

8 $${q}_{e}=\frac{RT}{{b}_{T}}ln{A}_{T}+\frac{RT}{{b}_{T}}ln{C}_{e}$$

*b*_*T*_ is the Temkin isotherm constant (J/mol), *R* is the universal gas constant (8.314 J/mol K), *T* is the temperature (*K*), and *A*_*T*_ is the Temkin isotherm equilibrium binding constant (l/g)^32^.

The fourth model examined to support the multilayer adsorption of iodine atoms onto the synthesized photocatalyst is the Harkins–Jura model, whose linear form (Eq. 9) is as follows^33^:

9 $$\frac{1}{{q}_{e}2}=\frac{B}{A}-\frac{1}{A}log{C}_{e}$$

where *A*_*H*_ and *B*_*H*_ are Harkins constants derived from the slope and intercept of the linear plots of *q*_*e*_^*2*^ versus log *C*_*e*_, respectively^33^.

#### Kinetics modeling

Adsorption kinetics provides data on adsorbent performance and mass transfer modes, such as diffusion, surface adsorption, intramolecular adsorption, and chemical adsorption. Adsorption kinetics are typically consistent with the pseudo-first-order kinetic model when the adsorption control factor is located in the boundary layer^5^. It indicates that a change in adsorption rate is proportional to the accessible sites on the surface of the adsorbent. As demonstrated by Eq. (10) ^8^:

10 $$\mathrm{ln}\left({q}_{e1}{-q}_{t}\right)=\mathrm{ln}{q}_{e1}{-k}_{1}t$$

where *q*_*e1*_ and *q*_*t*_ represent the quantity of adsorbate adsorbed at equilibrium time and time t, respectively (mg/g). *K*_*1*_ (1/min) is the pseudo-first-order rate constant^5^.

In addition, the pseudo-second-order kinetic model is described as follows (Eq. 11)^34^:

11 $$\frac{t}{{q}_{t}}=\frac{1}{{k}_{2}{q}_{e2}^{2}}+\frac{1}{{q}_{e2}}t$$

where *t* is the time (min) and *k*_*2*_ is the constant pseudo-second-order rate (g/mg min) ^34^.

The Elovich model is one of the most commonly employed kinetic models for analyzing the impact of temperature on adsorption systems. The expression for the Elovich model is (Eq. 12)^8^:

12 $${q}_{t}=\frac{1}{\beta }ln\left(\alpha \beta \right)+\frac{1}{\beta }lnt$$

where represents the initial adsorption rate constant (mg/g min) and Elovich's constant is the activation energy and surface coverage associated with chemisorption (g/m^2^)^8^.

According to the intraparticle diffusion model, the adsorption procedure consists of intraparticle diffusion, film diffusion, and emptying of the infill. This is crucial to the advancement of physical adsorption processes (Eq. 13), as it accounts for the formation of multiple layers based on van der Waals forces^35,36^:

13 $$ {\text{q}}_{{\text{t}}} = {\text{k}}_{{{\text{int}}}} {\text{t}}^{{{1}/{2}}} + {\text{C}} $$

where *k*_*int*_ represents the intra-particle diffusion rate constant (g/mg min) and the intercept of the plot and *C* represents the boundary layer effect or surface adsorption^35,36^.

#### Thermodinamics modeling

The Arrhenius equation determines the activation energy, *Ea*, which is the minimum amount of energy necessary to initiate a chemical reaction^5^.

14 $$ k_{app} = Ae^{{\frac{Ea}{{RT}}}} $$

15 $$ {\text{ln k}}_{{{\text{app}}}} = {\text{lnA}} - \frac{Ea}{{RT}} $$

where kapp represents the apparent rate constant (min^-1^), *Ea* is the activation energy (kJ mol^-1^), *T* is the temperature (K), *R* is the gas constant (8.314 J K^-1^ mol^-1^) and *A* is the Arrhenius constant (min^-1^)^22^. In the transition state theory (TST), the conventional Gibbs free energy, ΔG° (kJ mol^-1^), is defined by the following equations^6^:

16 $$ \Delta {\text{G}}^{^\circ } = - {\text{RTlnk}}_{{{\text{app}}}} $$

17 $$ \Delta {\text{G}}^{^\circ } = \Delta {\text{H}}^\circ - {\text{T}}\Delta {\text{S}}^\circ $$

where *ΔH°* is the standard enthalpy (kJ mol^-1^) and *ΔS°* is the standard entropy (JK^-1^ mol^-1^)^5^. TST implies the temperature dependence of the apparent rate constant in the Henry equation^33^:

18 $$ {\text{k}}_{{{\text{app}}}} = \frac{KBt}{h}{\text{exp}}\left( {\frac{\Delta G}{{RT}}} \right) $$

where *K*_*B*_ is the Boltzmann’s constant (*K*_*B*_ = 1.380510^–23^ JK^-1^) and *h* is the Planck’s constant (*h* = 1.380510^-23^Js)^33^.

19 $$ {\text{k}}_{{{\text{app}}}} = \frac{{k_{B} {\text{ T}}}}{h}{\text{exp}}\left( {\frac{\Delta S}{{RT}}} \right){\text{exp}}\left( {\frac{\Delta G}{{RT}}} \right) $$

20 $$\mathrm{ln}\left(\frac{{k}_{\mathrm{app}}}{{k}_{B}\mathrm{ T}}\right)=\frac{\Delta S^\circ }{R}- \frac{\Delta H^\circ }{RT}$$

The *ΔH*^*°*^ and frequency factor constant (*A*) are analogous to the activation energy quantity (*E*_*a*_)^6^.

21 $$\mathrm{A}=\frac{{k}_{B}\mathrm{ T}}{h}\mathrm{exp}\frac{\Delta S^\circ }{R}$$

22 $$ {\text{E}}_{{\text{a}}} = \Delta {\text{H}}^\circ + {\text{RT}} $$

#### Experimental design

Four independent variables were selected, including pH (**A**), photocatalyst dosage (**B**), exposure time (**C**), dye concentration (**D**), and US power (**E**) at three levels (Table 1). 48 experimental trials were prescribed by the CCD-RSM, and the response of independent variables was fitted to the model using the following regression equation (Table 2):^5^.

**Table 1:** Range levels of independent variables.

Independent variables	Range and level		
	− 1	0	+ 1
pH (**A**)	3	5	7
Photocatalyst dosage, mg (**B**)	25	62.5	100
Exposure time, min (**C**)	5	22.5	40
Concentarion of dye, mg/lit (**D**)	50	125	200
US power, W (**E**)	30	65	100

**Table 2:** ANOVA used to the designated quadratic model.

Source	Sum of Squares	df	Mean Square	F-value	p-value	
Model	4601.64	20	230.08	29.33	< 0.0001	Significant
**A**-pH	243.56	1	243.56	31.04	< 0.0001	
**B**-Exposure time	24.74	1	24.74	3.15	0.0871	
**C**-Concentraion dye	395.76	1	395.76	50.44	< 0.0001	
**D**-Photocatalyst dosage	59.56	1	59.56	7.59	0.0104	
**E**-US power	105.88	1	105.88	13.50	0.0010	
AB	18.00	1	18.00	2.29	0.1415	
AC	66.13	1	66.13	8.43	0.0073	
AD	45.13	1	45.13	5.75	0.0237	
AE	105.13	1	105.13	13.40	0.0011	
BC	21.13	1	21.13	2.69	0.1124	
BD	153.13	1	153.13	19.52	0.0001	
BE	120.13	1	120.13	15.31	0.0006	
CD	12.50	1	12.50	1.59	0.2177	
CE	2.00	1	2.00	0.2549	0.6177	
DE	72.00	1	72.00	9.18	0.0053	
A^2^	165.11	1	165.11	21.04	< 0.0001	
B^2^	207.97	1	207.97	26.51	< 0.0001	
C^2^	33.37	1	33.37	4.25	0.0489	
D^2^	8.22	1	8.22	1.05	0.3150	
E^2^	0.2604	1	0.2604	0.0332	0.8568	
Residual	211.84	27	7.85			
Lack of fit	168.50	22	7.66	0.8838	0.6268	Not significant
Pure error	43.33	5	8.67			
Cor total	4813.48	47				

23 $$Y={\beta }_{0}+\sum_{i=1}^{k}{\beta }_{i}{x}_{i}+ \sum_{i=1}^{k}{\beta }_{ii}{x}_{i}^{2}+ \sum_{i=1}^{k-1}\sum_{j=2}^{k}{\beta }_{ij}{x}_{i}{x}_{j}+ \varepsilon $$

where *β*_*0*_, *b*_*i*_, *b*_*ii*_, and *b*_*ij*_ are the intercept, linear, squared, and interaction coefficients, respectively. *Y* denotes the response. In addition, *x*_*i*_^*2*^*, x*_*j*_^*2*^*… x*_*k*_^*2*^ are the square effects, and *x*_*i*_*x*_*j*_*, x*_*i*_*x*_*k*,_ and *x*_*j*_*x*_*k*_ are the interaction effects of variables. *k* is the number of considered factors, and *ε* is the random errore^5^.

## Results and discussion

### Characterization

#### FTIR analysis

**Figure 1:**
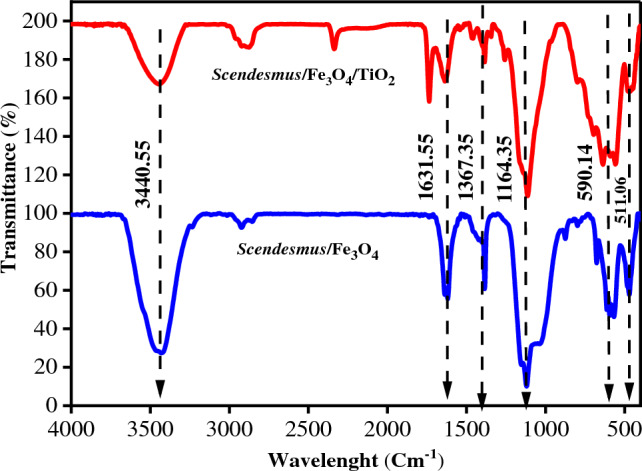
FTIR analysis of *Scendesmus*/Fe_3_O_4_ and *Scendesmus*/Fe_3_O_4_/TiO_2_.

As determined by FTIR spectroscopy, Fig. 1 depicts the functional groups of *Scendesmus*/Fe_3_O_4_ and *Scendesmus*/Fe_3_O_4_/TiO_2_. Strong –OH stretching is detected at 3440.55 cm^-1^ in *Scendesmus*/Fe_3_O_4_/TiO_2_ and scendesmus/Fe3O4, which may be related to the presence of water^6^. The bands at 1164.35 and 1367.35 cm^-1^ correspond, respectively, to the C=O, C–O, and Fe (C–O–Fe) stretching vibrations^22^. C=O at 1631.55 cm^-1^, however, indicated the presence of acetylacetonate on the photocatalyst surface^5^. The Fe–O content of *Scendesmus*/Fe_3_O_4_ and *Scendesmus*/Fe_3_O_4_/TiO_2_ is also confirmed by bands between 511.06 and 590.14 cm^-1^^37^. The observed bands between 550 and 900 cm^-1^ verify the presence of Ti–O–Ti and Fe–O^6^. In addition to Fe–O stretching vibrations, symmetric and asymmetric stretching, and COO^-^, the bands are also associated with Fe–O stretching vibrations. Anatase Fe–O and TiO_2_ are responsible for the peaks at 576 and 593 cm^-1^ in the *Scendemus*/Fe_3_O_4_/TiO_2_ spectrum^37^.

#### SEM,Mapping and VSM analysis

**Figure 2:**
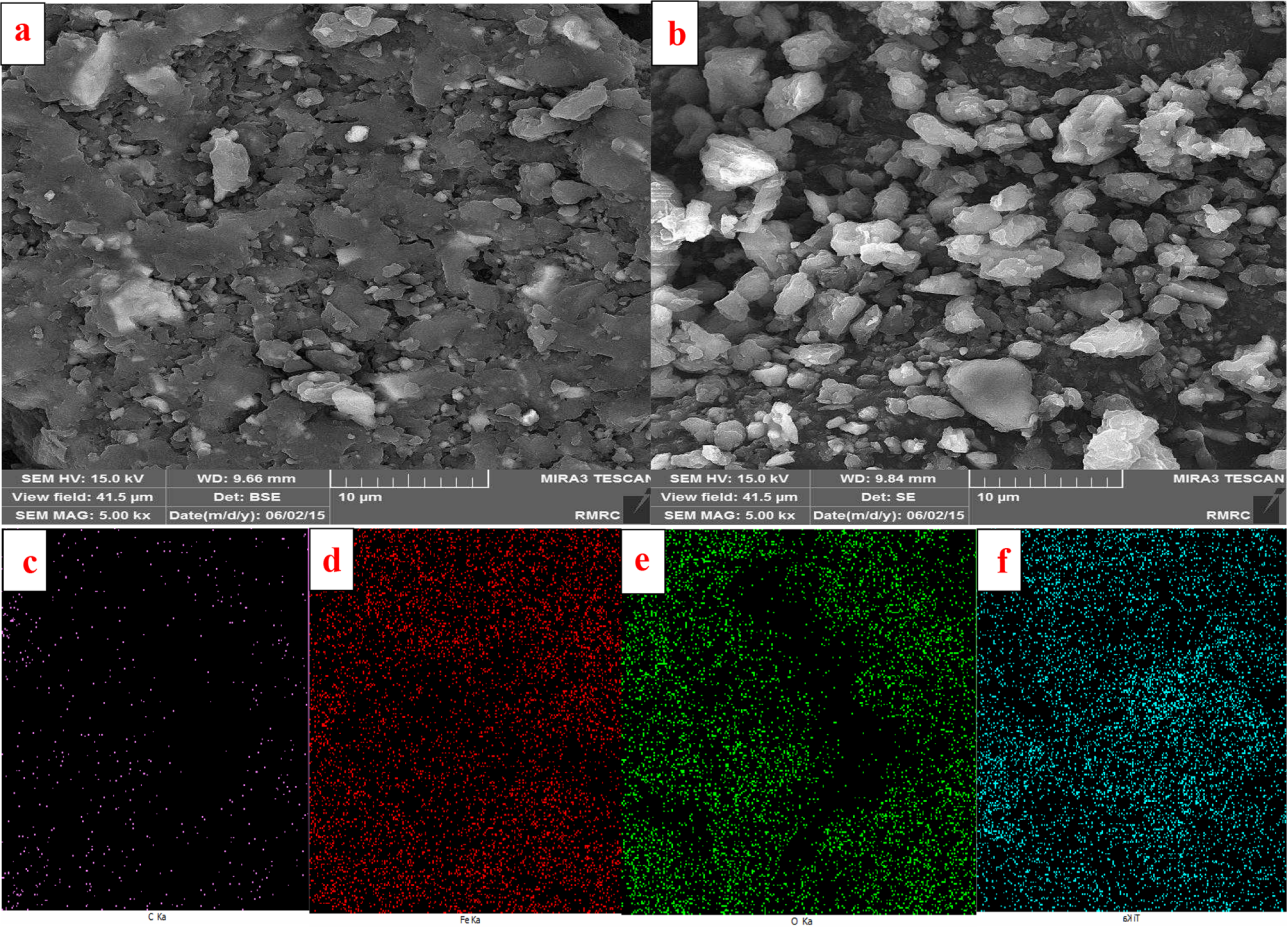
FESEM images *Scendesmus* (**a**) and *Scendesmus*/Fe_3_O_4_/TiO_2_ (**b**), Map analyses of the Scendesmus/Fe_3_O_4_/TiO_2_, (**c**) C ka, (**d**) Fe ka, (**e**) O ka, (**f**) Ti ka.

**Figure 3:**
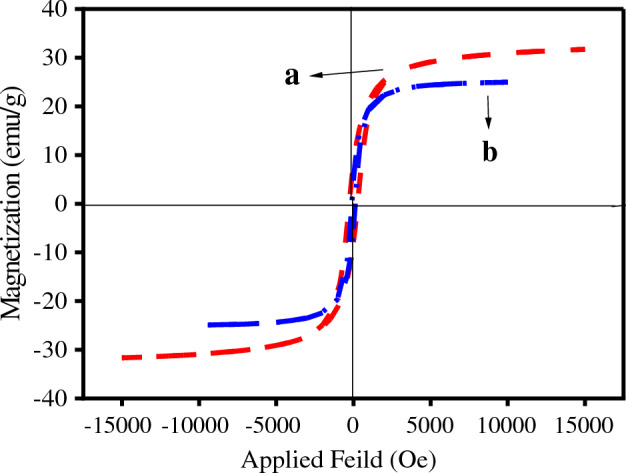
VSM analysis of (**a**) *Scendesmus*/Fe_3_O_4_ and (**b**) *Scendesmus*/Fe_3_O_4_/TiO_2_.

Figure 2a and b depict the surface morphology of *Scendesmus*/Fe_3_O_4_/TiO_2_, respectively. Figure 2a demonstrates the octagonal shape and flat surface of the *Scendesmus.sp*^37^. *Scendesmus* was uniformly coated with TiO_2_ particles for photocatalytic activity, as shown in Fig. 2b ^38^. This indicates that the titanium particles on the Scendesmus' surface are securely adhered. Therefore, the *Scendesmus*/Fe_3_O_4_/TiO_2_ synthesis was successful. Carbon, iron, oxygen, and titanium are present in the mapping analysis, confirming the formation of *Scendesmus*/Fe_3_O_4_/TiO_2_ (Fig. 2c–f)^39^. Based on VSM analysis, the presence of diamagnetic TiO_2_ in the structure of *Scendesmus*/Fe_3_O/TiO_2_ was responsible for a minor decrease in magnetic strength^5^. The photocatalyst exhibited robust magnetic activity against a 1.4 Tesla external magnet. In addition, the saturation magnetization (MS) value was approximately 16 emu/g, demonstrating the photocatalyst's high magnetic properties (Fig. 3)^22^.

### Central composite design

#### Model and statistical analysis

To design and optimize the interaction effects of five sono-photocatalytic process variables, the CCD model (RSM) was used. Table 2 displays the obtained results (actual and predicted responses) for the R195 degradation by means of the sono-photocatalytic process, after designating the number of trials using the CCD plan. Taking into consideration the coded factors, the proposed model equation (Eq. 24) was fitted to the quadratic model for R195 degradation following statistical analysis.

24 $$ \begin{aligned} {\text{Red195 Removal}}\left( \% \right) = & + {9}0.{57} - {5}.{\text{33 A}} + {1}.0{\text{6 B}} + {3}.{\text{22 C}} + {2}.{\text{39D}} + 0.0{\text{6 AB}} - 0.{\text{1875AC}} - {1}.{\text{19AD}} \\ & + {1}.0{\text{6BC}} + {1}.{\text{81BD}} + {1}.{\text{31CD}} - {19}.{\text{97A}}^{{2}} + 0.{\text{5263 B}}^{{2}} - 0.{\text{9737C}}^{{2}} - {1}.{\text{47 D}}^{{2}} \\ \end{aligned} $$

Table 3 presents a summary of the F-value, P-value, and R2 values used in the analysis of variance (ANOVA) to determine the significance and degree of fit of the employed model. The F-value and P-value for the degradation of R195 in the constructed model are 29.33 and 0.00001, respectively, indicating that the proposed model is significant^5^. As the factor with the highest F-value, the C-factor (dye concentration) is deemed significant. In addition, the P-value for lack of fit (LOF) reveals an insignificant mode (P-value > 0.05), which indicates a superior action compared to the model described^40^. Three categories of correlation coefficients, including R^2^ (0.9560), adjusted R^2^ (0.9234), and predicted R^2^ (0.8566), were used to evaluate the suitability of the models (Table 4).

**Table 3:** ANOVA results for Red195 removals.

Response	Sum of squares	Mean square	F Value	p-valuProb > F	Predicted R^2^
Red195 removal	4601.64	230.08	29.33	< 0.0001	0.8566
	Std.Dev	C.V	Ad. pre*	R^2^	Adj.R^2^
	2.80	3.35	20.63	0.9560	0.9234

**Table 4:** Experimental conditions, and the obtained results.

Run	Value						
	A	B	C	D	E	R195 removal (%)	Predicted removal(%)
1	7 (+ 1)	40 (+ 1)	50 (− 1)	25 (− 1)	30 (− 1)	71	71.85
2	7 (+ 1)	5 (− 1)	50 (− 1)	100 (+ 1)	100 (+ 1)	86	83.69
3	3 (− 1)	40 (+ 1)	200 (+ 1)	25 (− 1)	100 (+ 1)	82	79.03
4	7 (+ 1)	40 (+ 1)	50 (− 1)	25 (− 1)	100 (+ 1)	81	79.38
5	3 (− 1)	5 (− 1)	50 (− 1)	25 (− 1)	100 (+ 1)	80	81.02
6	3 (− 1)	40 (+ 1)	50 (− 1)	100 (+ 1)	100 (+ 1)	85	88.50
7	5 (0)	22.5 (0)	125 (0)	62.5 (0)	65 (0)	100	97.10
8	7 (+ 1)	40 (+ 1)	50 (− 1)	100 (+ 1)	30 (− 1)	77	77.00
9	3 (− 1)	40 (+ 1)	50 (− 1)	25 (− 1)	30 (− 1)	84	81.83
10	7 (+ 1)	5 (− 1)	200 (+ 1)	100 (+ 1)	100 (+ 1)	70	74.12
11	7 (+ 1)	5 (− 1)	200 (+ 1)	25 (− 1)	30 (− 1)	73	68.44
12	7 (+ 1)	40 (+ 1)	200 (+ 1)	25 (− 1)	100 (+ 1)	70	70.55
13	5 (0)	22.5 (0)	125 (0)	62.5 (0)	30 (− 1)	94	95.66
14	7 (+ 1)	5 (− 1)	200 (+ 1)	100 (+ 1)	30 (− 1)	70	67.34
15	3 (− 1)	5 (− 1)	50 (− 1)	100 (+ 1)	30 (− 1)	80	80.14
16	3 (− 1)	5 (− 1)	200 (+ 1)	25 (− 1)	100 (+ 1)	74	74.70
17	7 (+ 1)	5 (− 1)	50 (− 1)	25 (− 1)	100 (+ 1)	80	81.30
18	5 (0)	22.5 (0)	125 (0)	62.5 (0)	65 (0)	98	97.10
19	7 (+ 1)	40 (+ 1)	200 (+ 1)	100 (+ 1)	30 (− 1)	70	69.67
20	3 (− 1)	5 (− 1)	50 (− 1)	25 (− 1)	30 (− 1)	90	88.50
21	5 (0)	5 (− 1)	125 (0)	62.5 (0)	65 (0)	85	87.07
22	5 (0)	22.5 (0)	125 (0)	25 (− 1)	65 (0)	97	97.60
23	3 (− 1)	40 (+ 1)	200 (+ 1)	100 (+ 1)	100 (+ 1)	87	87.93
24	7 (+ 1)	5 (− 1)	50 (− 1)	100 (+ 1)	30 (− 1)	76	77.91
25	3 (− 1)	40 (+ 1)	200 (+ 1)	25 (− 1)	30 (− 1)	76	77.75
26	5 (0)	40 (+ 1)	125 (0)	62.5 (0)	65 (0)	90	88.78
27	5 (0)	22.5 (0)	125 (0)	62.5 (0)	65 (0)	98	97.10
28	5 (0)	22.5 (0)	125 (0)	62.5 (0)	65 (0)	92	97.10
29	7 (+ 1)	5 (− 1)	50 (− 1)	25 (− 1)	30 (− 1)	80	81.52
30	5 (0)	22.5 (0)	125 (0)	62.5 (0)	65 (0)	98	97.10
31	3 (− 1)	5 (− 1)	200 (+ 1)	100 (+ 1)	30 (− 1)	73	75.32
32	3 (− 1)	40 (+ 1)	50 (− 1)	25 (− 1)	100 (+ 1)	80	82.11
33	3 (− 1)	5 (− 1)	200 (+ 1)	25 (− 1)	30 (− 1)	80	81.17
34	5 (0)	22.5 (0)	200 (+ 1)	62.5(0)	65 (0)	86	90.01
35	3 (− 1)	40 (+ 1)	50 (− 1)	100(+ 1)	30 (− 1)	82	82.22
36	3 (− 1)	5 (− 1)	50 (− 1)	100(+ 1)	100 (+ 1)	80	78.67
37	7 (+ 1)	5 (− 1)	200 (+ 1)	25 (− 1)	100 (+ 1)	70	69.22
38	7 (+ 1)	40 (+ 1)	200 (+ 1)	100(+ 1)	100 (+ 1)	85	84.20
39	3 (− 1)	40 (+ 1)	200 (+ 1)	100(+ 1)	30 (− 1)	83	80.65
40	7 (+ 1)	22.5 (0)	125 (0)	62.5(0)	65 (0)	85	86.25
41	5 (0)	22.5 (0)	125 (0)	62.5(0)	65 (0)	100	97.10
42	3 (− 1)	22.5 (0)	125 (0)	62.5(0)	65 (0)	92	91.60
43	7 (+ 1)	40 (+ 1)	200 (+ 1)	25 (− 1)	30 (− 1)	60	62.02
44	7 (+ 1)	40 (+ 1)	50 (− 1)	100(+ 1)	100 (+ 1)	91	90.52
45	5 (0)	22.5 (0)	50 (− 1)	62.5(0)	65 (0)	100	96.84
46	5 (0)	22.5 (0)	125 (0)	62.5(0)	100 (+ 1)	100	*99.19*
47	5 (0)	22.5 (0)	125 (0)	100(+ 1)	65 (0)	100	100.25
48	3 (− 1)	5 (− 1)	200 (+ 1)	100(+ 1)	100 (+ 1)	78	74.85

**Figure 4:**
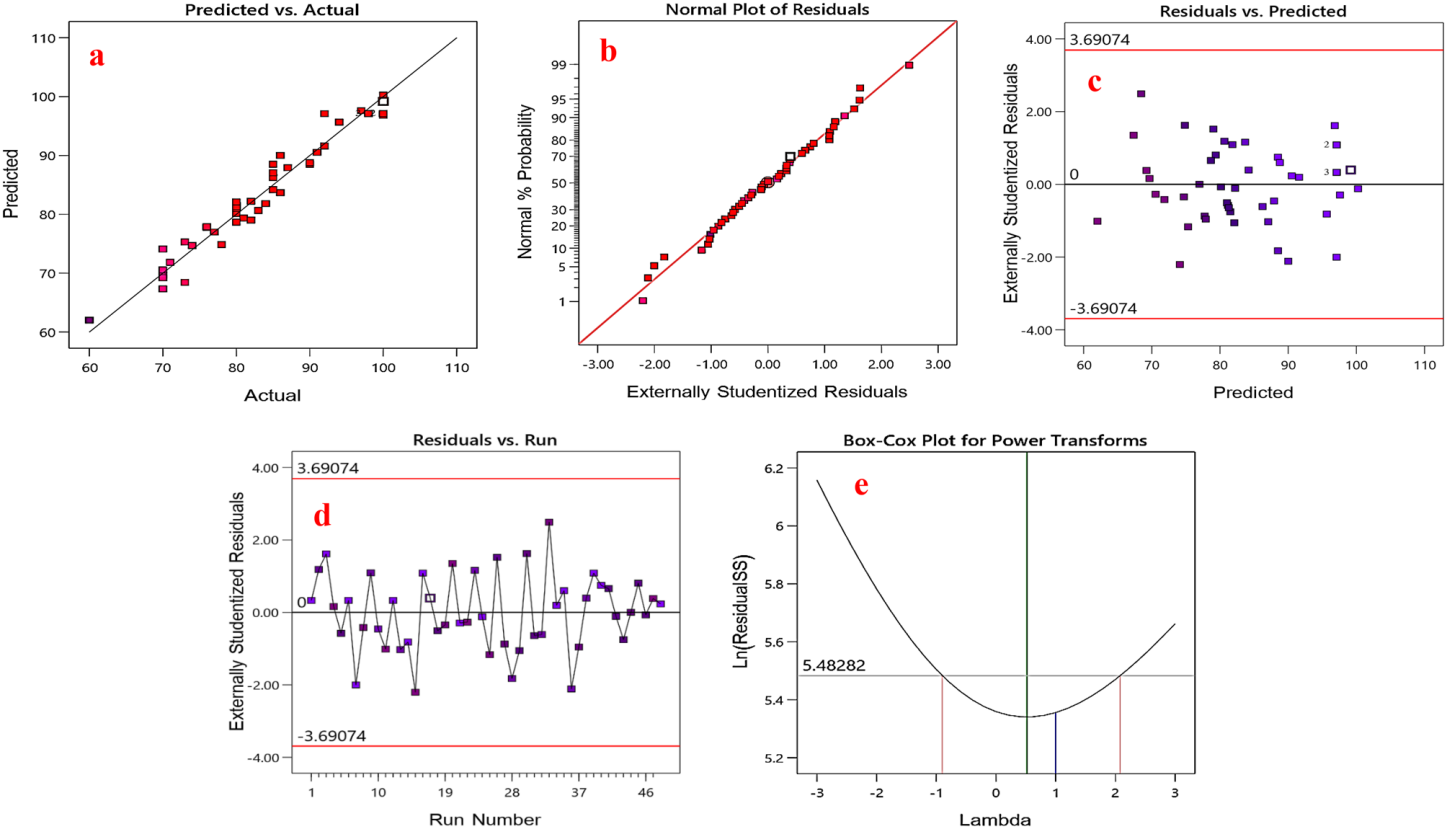
The diagnostics plots for validation of quadratic model: (**a)** predicted values vs. factual values, (**b**) normal probability distribution of residuals, (**c)** internally studentized residuals vs. predicted values plot, (**d**) internally studentized residuals vs. run numbers, and (**e)** Box-Cox plot.

Figure 4a was constructed from a comparison of predicted values against factual values^41^. The validity of the model was confirmed by a straight trend line connecting residuals (Fig. 4b). Comparing the residual's plot to the predicted values (Fig. 4c) and run numbers (Fig. 4d) requires a random dispersion. A Box-Cox procedure (Fig. 4e) was used to determine the normality of the data and establish the model significance of the sonophotocatalytic process^42^.

#### The effect of single factors

The variance analysis (ANOVA) for R195 removal is presented in Table 3. Among pH, exposure time, photocatalyst dosage, R195 concentration, and US power as a single variable, each factor with a high mean square and high F-value exerts the greatest influence on the sono-photodegradation of R195. Thus, the effect of variables on R195's degradation is^43^:

$$ {\text{Concentration of Red}}195 \le  {\text{pH}} \le {\text{US Power}} \le {\text{otocatalyst dosage}} \le {\text{Exposure time}}. $$

The concentration of R195 with a mean square and F-value of 230.08 and 29.33, respectively, was the most crucial factor in R195 removal^5^.

#### Three dimensional response surface schemes

**Figure 5:**
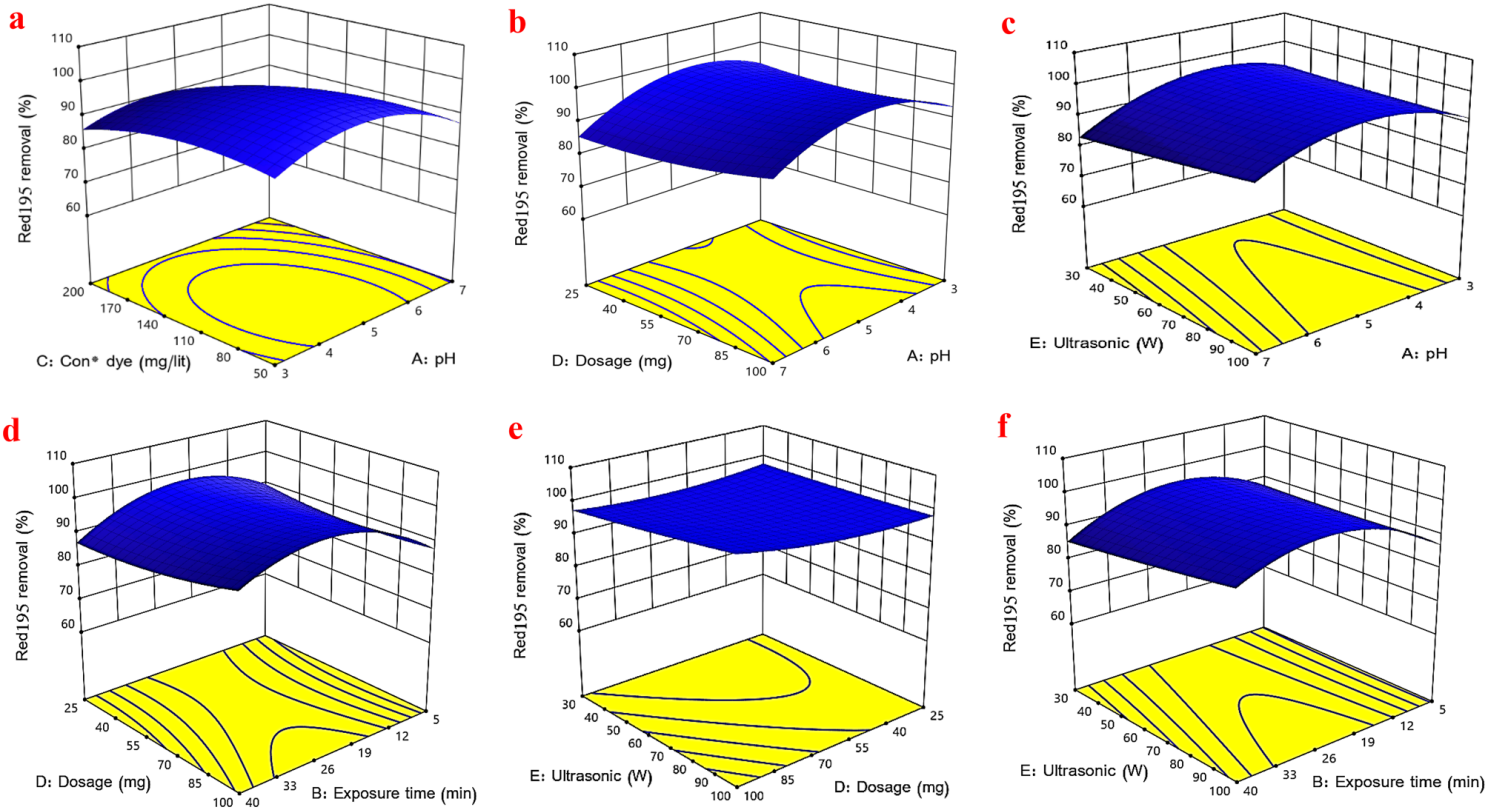
The three-dimensional interaction effects of pH and concentration of dye (**a**), pH and photocatalyst dosage (**b**), pH and US power (**c**), exposure time and photocatalyst dosage for (**d**), photocatalyst dosage and US power (**e**), exposure time and US power (**f**).

Three-dimensional (3D) graphical schemes (Fig. 5) were implemented in order to assess the individual and combined effects of operational variables on the sonophotocatalytic degradation of R195. Figure 5a depicts the simultaneous effect of initial pH (three to seven) and dye concentration (fifty to two hundred mg/lit) on optimization under constant conditions (exposure time = 22 min, US power = 65W, and photocatalyst dosage = 62.50 mg). Maximum removal efficacy was observed in a medium with a pH of 5 after 15 min. This trend can be attributed to the following elements: (a) A test apparatus operating under identical conditions will produce analogous results (equal production of hydroxyl radicals at each initial R195 concentration)^44^; (b) the generation of hydroxyl radicals is proportional to the initial concentration of R195^40^. Therefore, an increase in the number of dye molecules can lead to the saturation of active sites on the surface of the photocatalyst and inadequate production of hydroxyl radicals^42^. (c) Produced intermediates of R195 during sonophotocatalytic degradation (at high concentrations of R195) reduce removal efficiency due to a competition reaction between the dye and intermediate with free radicals^5^. Dye molecules and photocatalyst nanorods are brought into favorable contact by increasing the number of accessible active sites. According to the scientific literature, the pka of R195 (3.6) and the pHpzc of TiO_2_ (6.8) remind us that electrostatic interaction between the negatively charged facet of R195 (at pH > 3.6) and the positively charged facet of nanorods (at pH 6.8) is the primary reason for the accelerated degradation of R195^6,22^. Additionally, by undertaking sonophotocatalytic reactions in an acidic medium and extending the duration of US irradiation, residual R195 in the cavitation bubbles containing more hydroxyl radicals can provide a greater potential for oxidation of the model pollutant^44^. Amri et al.^45^ and Nangia et al.^46^ also reported similar results with our findings^45,46^. 5b depicts the reciprocal effect of pH and photocatalyst concentration. By increasing the pH (from 3 to 7) and decreasing the amount of catalyst (from 100 to 25 mg), the removal efficiency was diminished. At a higher photocatalyst dosage, more cavitation bubbles may be produced in the region of ultrasound irradiation, resulting in sufficient free radical generation^22^.

Figure 5c investigates the effects of pH and US power on the elimination of R195 by varying the US power from 30 to 100 W and the pH from 3 to 7. As the US power increased from 30 to 100 W, the percentage of removal rose from 55 to 85%.The increase in dye degradation can be attributed to a number of factors, including the disintegration of dye molecules, the destruction of dye structures as a result of an increase in energy pressure, and the total decomposition of dye^5,40,47^. Figure 5d depicts the interaction effect of contact time and photocatalyst dose. The duration ranged from 5 to 40 min, and the photocatalyst dose ranged from 25 to 100 mg. The removal percentage rose from 65 to 98% as the photocatalyst dosage increased. The optimal dose for reducing photocatalyst consumption was determined to be 62 mg. In relation to the time variable, the photocatalytic removal rate increased from 5 to 83% between 5 and 22 min, and then decreased from 83 to 75% between 22 and 40 min. Increasing the photocatalyst's contact time with US and UVA lamps initially accelerated the oxidation processes. After 22 to 40 min, the removal rate decreased due to increased competition between the dye molecule and the photocatalyst and the saturation of the active sites^5^. Thus, 22 min was considered the optimal duration. Figure 5e examines the effect of the US power parameter in the range of 30 to 100 W and the photocatalyst dose in the range of 25 to 100 mg. Both parameters increased directly in proportion to the amount of dye removed. Figure 5f depicts the interaction effect of photocatalyst dose and US power. Both parameters increased directly in proportion to the amount of dye removed. The dye removal percentage increased from 64 to 95% when the photocatalyst dose was increased from 25 to 100 mg, and when the US power was increased from 30 to 100W. It is due to the high availability of active reaction sites, OH radicals, and electron–hole pairs^8,43,46^.

### Mechanism of sonophotocatalytic degradation

To identify reactive active species in the degradation of R195 by the *Scendesmus*/Fe_3_O_4_/TiO_2_ UV/US process, radical trapping experiments with a variety of scavengers were conducted. 100 percent (reactor without scavenger) to 86.9 percent (reaction solution with TiO_2_ present). This event demonstrates that O_2_ molecules were converted into an O_2_^•-^reaction solution, decreasing the effectiveness from one hundred percent to 66.7%^48^. These findings indicate that the sonophotocatalytic process generates •OH, h^+^, and O_2_^•-^ in the reaction solution^49^. A potential degradation mechanism for the sonophotocatalytic process has been postulated based on the results of the entrapment test and previous research^6^. Using adsorption and photolysis, certain contaminants can be eliminated. Sonolysis and sonophotocatalytic processes can also be utilized to eliminate R195 dye in solution and on a catalyst's surface. During these processes, the heated spot event (resulting from the cavitation effect) can separate water molecules into •OH and H• (Eq. (25))^6,22,48^. US radiation, via the sonoluminescence mechanism, produces visible light radiation for the generation of electron/hole pairs in the valence and conduction bands of particles along the same pathway^28^. Under UV radiation, however, *Scendesmus*/Fe_3_O_4_/TiO_2_ nanoparticles generate electron–hole pairs by absorbing light (Eq. (26))^50^. In addition to directly degrading the contaminant, the holes in the valence band can decompose H_2_O molecules and hydroxyl ions (OH^-^) to generate ^•^OH (Eqs. (27) and (28))^6^. All species generated in the solution and solid phases are capable of transforming pollutants into CO_2_, H_2_O, and biodegradable products (Eq. (29))^22^.

25 $$ {\text{H}}_{2} {\text{O + )))}} \to \cdot {\text{OH}} + {\text{H}}\cdot $$

26 $$ Scendesmus/{\text{Fe}}_{{3}} {\text{O}}_{{4}} /{\text{TiO}}_{{2}} + ))) + {\text{UVA}} \to {\text{h}}_{{{\text{VB}} + }} + {\text{e}}_{{{\text{CB}}^{ - } }} $$

27 $$ {\text{h}}_{{{\text{VB}} + }} + {\text{H}}_{{2}} {\text{O}} \to {\text{H}}^{ + } + \cdot {\text{OH}} $$

28 $$ {\text{h}}_{{{\text{VB}} + }} + {\text{OH}}^{ - } \to \cdot {\text{OH}} $$

29 $$ {\text{R195}} + {\text{ active radicals}} \to {\text{CO}}_{{2}} + {\text{H}}_{{2}} {\text{O }} + {\text{ byproducts}} $$

### Isotherm, kinetcs and thermodinamics model

**Figure 6:**
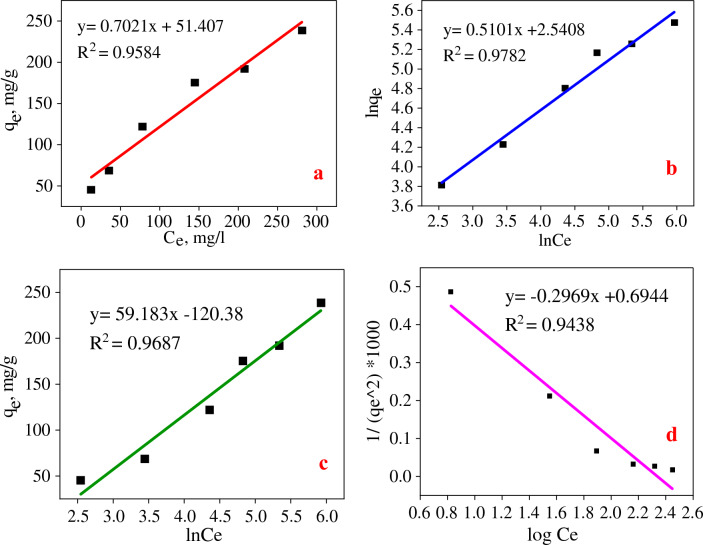
Langmuir (**a**), Freundlich (**b**), Temkin (**c**), and Harkins–Jura (**d**) isotherm models for R195 removal.

**Figure 7:**
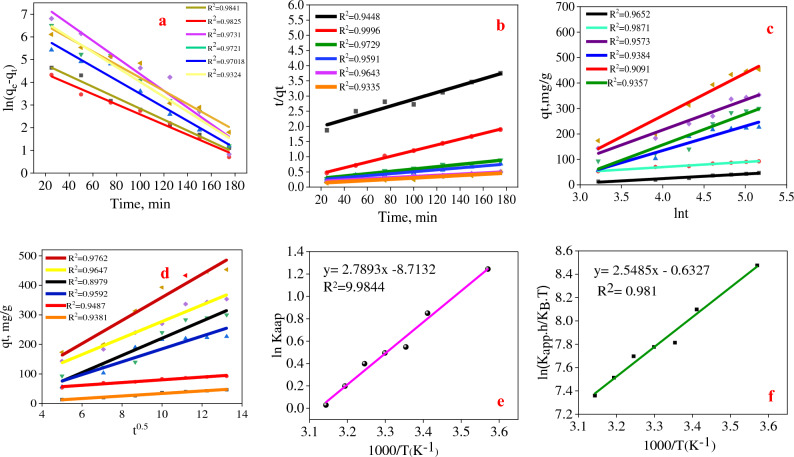
Pseudo-first-order (**a**), pseudo-second-order (**b**), and Elovich (**c**) and Intra particle diffusion (d) kinetic models for the R195 concentrations of 50 ( ), 100 ( ), 200 ( ), 300 ( ), 400 ( ), and 500 ( ) mg/100 ml. Enthalpy and entropy change for the photocatalytic decomposition of R195, (**e**) Arrhenius plot and (**f**) Henry Eyring plot.

As depicted in Fig. 6, the Langmuir, Freundlich, Temkin, and Harkins–Jura isotherm adsorption models were implemented. The Freundlich (R^2^ = 0.9782), Harkins Jura (R^2^ = 0.9438), Temkin (R^2^ = 0.9687), and Langmuir (R^2^ = 0.9584) models (Table 5) provided a more accurate description of the equilibrium adsorption data. Freundlich's isotherm is an empirical model that depicts photocatalyst-pollutant interactions on heterogeneous surfaces and in multilayer adsorption^5^. With 1/n and K_F_ values of 0.4036 and 12.68 mg1^-n^ g^-1^ L^-n^, respectively, the adsorption process and reaction's intensity were favorable. In addition, the Harkins–Jura model confirmed the adsorption of R195 molecules onto the heterogeneous surface^33^. In contrast, the presence of exothermic and physical adsorption between R195 molecules and Scendesmus, Fe_3_O_4_, and TiO2 was indicated by the positive value of b_T_ (43.266 J/mol) in the Temkin isotherm^32^. The optimal adsorption capacity (q_m_) predicted by the Langmuir isotherm was close to the experimental value, indicating that the photocatalyst had a uniform surface with the same activation energy^31^. In addition, the R_L_ value between 0 and 1 (R_L_ = 0.0269) indicated that the adsorption procedure was successful. Using pseudo-first-order, pseudo-second-order, Elovich, and intra-particle diffusion kinetic models, the R195 removal mechanism was predicted^5^. According to Fig. 7 and Table 5, the equilibrium condition for R195 removal was stabilized after 60 min for all concentrations tested, ranging from 50 to 500 mg/l, as shown in Fig. 7 and in Table 5. At 300 mg/l of R195, the empirical data were best described by the pseudo-first-order (PFO) model with R^2^ = 0.9925. These findings reveal a correlation between the quantity of physical R195 adsorption and the increased driving force responsible for the efficient diffusion of R195 molecules to the surface of *Scendesmus*/Fe_3_O_4_/TiO_2_ and their occupation of the remaining active sites^33^. The pseudo-second-order (PSO) model best described the adsorption process of 100, 200, and 400 mg/l of initial R195 concentrations, with R^2^ values of 0.9983, 0.9731, and 0.9722, respectively, when considering chemisorption as the predominant adsorption process with electron donor–acceptor and dispersal interactions^51^. The Elovich model also demonstrated the chemical adsorption of R195 molecules on the solid surfaces of exceedingly heterogeneous adsorbents (R2 > 0.9871)^5^. The intra-particle diffusion model provided the best fit to the adsorption data (Table 5, R^2^ = 0.9857 for 50 mg/l R195 and R^2^ = 0.9398 for 500 mg/l R195, respectively). Since the relationship between qt and t0.5 is linear, intra-particle diffusion should be the rate-regulating process ^35,36^.

**Table 5:** Parameters of isotherm, kinetics and thermodynamics model.

Kinetic	Parameters/Value			
Pseudo-first-order	K_1_ (min^-1^)	q_e_ (mg/g)	R^2^	
50	− 0.0251	241.815	0.9812	
100	− 0.0263	153.394	0.9925	
200	− 0.0289	664.230	0.9731	
300	− 0.0338	1738.095	0.9752	
400	− 0.0374	3385.488	0.9352	
500	− 0.0295	1487.73	0.9324	

Pseudo-second-order	K_2_ (g.mg^-1^ min^-1^)	q_e_ (mg/g)	R^2^	
50	4604.777	90.29	0.9557	
100	44,976.76	107.29	0.9983	
200	53,273.72	323.52	0.9731	
300	33,598.76	276.26	0.9589	
400	18,786.43	537.32	0.9722	
500	26,125.04	600.01	0.9424	

Elovich	α (mg g^-1^ min^-1^)	β (g/mg)	R^2^	
50	1.396	0.056	0.9652	
100	12.976	0.052	0.9871	
200	22.922	0.019	0.9573	
300	11.121	0.008	0.9384	
400	14.004	0.007	0.9091	
500	273.723	0.005	0.9347	

Intra particle diffusion	C (mg/g)	K_int_ (mg.g^- 1.min-0.5)^	R^2^	
50	− 8.8121	4.3797	0.9857	
100	− 32.921	4.7259	0.9623	
200	− 33.598	13.942	0.8869	
300	− 44.076	28.138	0.9583	
400	− 23.943	29.758	0.9494	
500	− 31.195	40.165	0.9398	

Isotherm	Parameters	Value		
Langmuir	*q*_m_ (mg/g)	1.42		
	b (l/mg)	36.09		
	R^2^	0.9584		
	R_L_	0.0269		
Freundlich	K_F_ (mg^1-n^ g^-1^ L^-n^)	12.68		
	1/n	0.4036		
	R^2^	0.9782		
Harkins–Jura	A	+ 3.368		
	B	2.338		
	R^2^	0.9438		
Temkin	b_T_ (J/mol)	43.26		
	A_T_ (l/g)	0.1308		
	R^2^	0.9687		

T, K	*K*_app_(*10^–3^), min^-1^	Ln(*K*_aap_h/K_B_T)	ΔG^o^, KJ mol^-1^	
Transition theory				
288.15	0.3293	− 48.4941	− 105.69	
293.15	0.4300	− 48.2445	− 457.192	
298.15	0.5546	− 48.0069	− 1287.11	
303.15	0.6066	− 47.9340	− 2099.44	
308.15	0.7799	− 47.6990	− 3241.03	
313.15	0.8146	− 47.6716	− 3852.97	
318.15	0.8666	− 47.6256	− 4948.46	

ΔH^o^, KJ mol^-1^	ΔS^o^, KJ mol^-1^ k	Ea, kJ mol^-1^	A, s^-1^	R^2^
Transition theory				
− 25.368	+ 21.167	− 69.121	7.151	0.9810

Ea, kJ mol^-1^	A, s^-1^	R^2^		
Arrhenius theory				
+ 72.414	37.326	0.9844

As shown in Table 5, the activation energy was calculated to be 69.12 kJ mol^-1^. The exothermic adsorption mechanism was revealed by the negative standard enthalpy of activation (ΔH^o^ = −23.368)^5^. Moreover, the increasing value of the standard Gibbs free energy of activation (ΔG^o^) with increasing temperature demonstrated that the process was not spontaneous^22^. The positive value of standard entropy of activation (ΔS^o^ =  + 21.167) was related to the transient molecular configuration at the summit of the energy barrier, indicating an increase in the degree of loss of freedom when the activated complex is formed from the reactants^6,33^.

### Potential scendesmus/Fe_3_O_4_/TiO_2_ recycle

**Figure 8:**
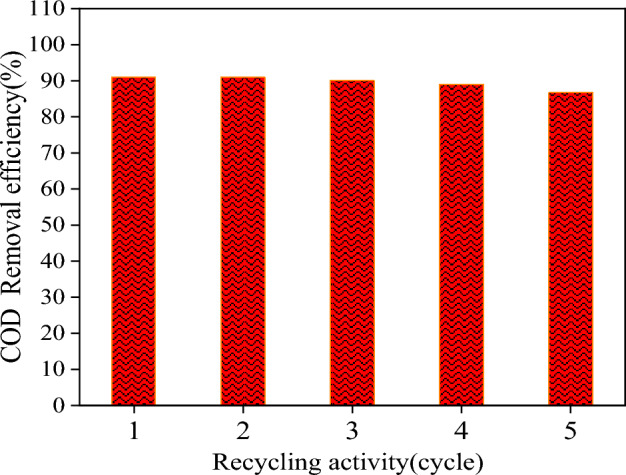
The reusability of the *Scendesmus*/Fe_3_O_4_/TiO_2_ within five cycles.

Under constant conditions (pH = 5, photocatalyst dosage = 100 mg, initial R195 concentration = 100 mg/l, ultrasound power = 38W, and exposure time = 20 min), the reusability of the synthesized *Scendesmus*/Fe_3_O_4_/TiO_2_ photocatalyst was evaluated (Fig. 8). After five cycles, the photocatalytic efficiency of *Scendesmus*/Fe_3_O_4_/TiO_2_ remained above 95% without any discernible decline. Due to its stability, the synthesized *Scendesmus*/Fe_3_O_4_/TiO_2_ composite is economically viable for use in the effluent treatment process^22^. Future product development and convincing investors and stakeholders could increase the renewability and cost-effectiveness of the final photocatalyst^6^ .

### Comparison of sonophotocatalytic degradation with other methods

Numerous techniques for degrading textile dyes have been investigated. Table 6 compares these techniques for the degradation of R195. The comparison demonstrates that sonophotocatalysis of R195 using *Scendesmus*/Fe_3_O_4_/TiO_2_ as a photocatalyst yields superior results in comparison to other techniques. According to the obtained data, the present photocatalyst (*Scendesmus*/Fe_3_O_4_/TiO_2_) is novel, relatively effective, cost-effective, and broadly accessible. *Scendesmus* was used as a precursor for the first time in this study to produce a magnetic photocatalyst and eliminate R195 dye. In this investigation, garbage was used as a photocatalyst to remove pollutants from farm effluent. From an ecological standpoint, this matter is of the utmost importance.

**Table 6:** Comparison with various Methods for the Removal of R195 Dyes.

Dye	Method	Time(min)	Degredation(%)	Refrencess
Rhodamine B (RhB)	photocatalytic removal	35	84.5	^52^
Methyl red (MR)	Adsorption	60	85	^53^
Rhodamine B (RhB)	photocatalytic removal	30	73	^54^
black 5	membrane	40	70.7	^55^
Red195	adsorption- photodegradation	35	89.5	^56^
Red195	ultrafiltration membranes	40	88	^57^
yellow dyes	photodegration	60	91	^58^
Methylene blue	Biodegration	60	85	^59^
Methylene blue	photodegration	75	85	^60^
Methylene blue	Photodegration–visible light	120	90	^61^
Organic dye	Sonophotocatalyst	20	98	^13^
Red195	Sonophotocatalyst	20	92	^20^
Rhodamine b	Sonophotocatalyst	240	92	^19^
Acid red 14	Sonophotocatalyst	21	100	^49^
Victoria blue	Sonophotocatalyst	60	71.97	^18^
Yellow AB and Remazol brilliant Violet-5R	Sonophotocatalyst	30	95.5–88.9	^17^

## Conclusion

In the present investigation, *Scendesmus*/Fe_3_O_4_/TiO_2_ was synthesized as a novel sonophotocatalyst for degrading R195 dye. Diagnostic analyses such as SEM, mapping, FTIR, and VSM confirmed the efficacy of the TiO_2_ coating on *Scendesmus*/Fe_3_O_4_ and the resulting photocatalyst. *Scendesmus*/Fe_3_O_4_/TiO_2_ nanoparticles are effective against sonolysis, photolysis, adsorption, and photocatalytic processes when exposed to ultraviolet light and ultrasonic waves. The R195 removal efficiency increases as operating parameters such as photocatalyst dosage, ultrasound power, radiation power, and exposure time are increased, but decreases as initial pH and initial R195 concentration are increased*.* Under optimal conditions, such as a pH of 5, a photocatalyst dosage of 62.5 mg/l, an exposure duration of 22 min, an initial R195 concentration of 125 mg/m3, and a US power of 65W, the maximum R195 removal was 100%. According to the proposed quadratic model, the concentration of R195 was the most significant variable because of its high F-value. The Freundlich isotherm model and the intra-particle diffusion model best fit the experimental data when taking into account the heterogeneous surface of the photocatalyst and multilayer adsorption. Thermodynamic analysis has confirmed that endothermic and nonspontaneous adsorption processes occur. Under repeated irradiation from the United States, the recycled *Scendesmus*/Fe_3_O_4_/TiO_2_ demonstrates exceptional activity and regular stability for pesticide removal. The addition of different scavengers to evaluate the active species of the sonophotocatalytic process revealed that all three species, O_2_, ^•^OH, and h^+^, were produced during the process and that h^+^ is the active species in the degradation of R195 into CO_2_ and H_2_O. The *Scendesmus*/Fe_3_O_4_/TiO_2_ photocatalyst was deemed a novel sonophotocatalyst for the mineralization of R195 based on analyses of various parameters and stability experiments. In conclusion, the transformation of scendesmus into a photocatalyst can not only eliminate the need for additional R195 degradation and reduce the cost of wastewater treatment, but also provide a valuable and efficient photocatalyst at a lower cost than commercial Microalgae that contributes to environmental preservation.

## Data Availability

The data and materials from the current study are available from the corresponding author on reasonable request.
